# Clinical factors associated with mortality in pediatric seawater drowning: a 13-year emergency department experience from a Turkish coastal province

**DOI:** 10.3389/fped.2026.1901281

**Published:** 2026-08-04

**Authors:** Şeyma Şimşirgil Kara, Teoman Erşen, Özhan Özcan, Sema Tural Bozoğlu, Dilek Sağır, Kıvanç Öncü

**Affiliations:** 1 Faculty of Health Sciences, Sinop University, Sinop, Türkiye; 2 Department of Emergency Medicine, Sinop Atatürk State Hospital, Sinop, Türkiye; 3 Department of Surgery and Critical Care, Sinop Atatürk State Hospital, Sinop, Türkiye; 4 Department of Pediatric Surgery, Sinop Atatürk State Hospital, Sinop, Türkiye; 5 Department of Anesthesiology and Reanimation, Sinop Atatürk State Hospital, Sinop, Türkiye

**Keywords:** drowning, emergency medicine, epidemiology, mortality, pediatrics, prognosis, seawater, Szpilman score

## Abstract

**Introduction:**

Pediatric drowning remains a leading cause of preventable childhood mortality, yet epidemiological and clinical data from coastal regions of Türkiye are scarce.

**Methods:**

This retrospective cross-sectional study analyzed all pediatric seawater drowning cases (ages 0-18 years) admitted to a referral emergency department in a Turkish coastal province between July 2011 and July 2024, with the aim of describing demographic and clinical characteristics and identifying factors associated with in-hospital mortality.

**Results:**

A total of 66 patients were included (mean age 10.64 ± 4.86 years; 60.6% male). The overall mortality rate was 10.6%. Over 90% of cases occurred in July or August, and nearly half involved non-resident visitors, reflecting the region's strong seasonal tourism pattern. Non-survivors were characterized by a Glasgow Coma Scale score of 3 and a median Szpilman score of 6 on admission, compared to scores of 15 and 1, respectively, among survivors (*p* < 0.001). Significant laboratory differences between non-survivors and survivors included markedly lower arterial pH and bicarbonate, and higher pCO₂, lactate, creatinine, transaminases (AST, ALT), lactate dehydrogenase, sodium, and potassium levels (all *p* < 0.05). All non-survivors received cardiopulmonary resuscitation and required endotracheal intubation, while none of the survivors required resuscitation. Radiological lesions showed a non-significant trend toward higher prevalence in non-survivors.

**Discussion:**

These findings indicate that neurological status on arrival, Szpilman score, arterial blood gas parameters, and specific biochemical markers may serve as early indicators associated with mortality to support risk stratification in pediatric drowning. Region-specific prevention strategies targeting adolescents and seasonal visitors, combined with rapid prehospital response, remain critical to reducing drowning-related mortality.

## Introduction

Despite its preventable nature, pediatric drowning remains a significant contributor to childhood morbidity and mortality (1, 2). While numerous international studies have described the epidemiology and acute management of drowning (3, 4), research specifically addressing Türkiye's pediatric population—particularly in coastal provinces where seasonal tourism and seawater exposure are common—remains limited (5). By addressing this critical knowledge gap, our study aims to inform both clinical decision-making and regionally tailored public health strategies.

Pediatric drowning is recognized globally as one of the leading causes of preventable morbidity and mortality in childhood (1, 2). Recent estimates suggest that children account for a disproportionately high number of drowning-related deaths, particularly in low- and middle-income countries (6). The incidence and outcome of drowning are shaped by multiple factors, including geographic and environmental conditions, socioeconomic background, and child-specific behavioral traits. Children aged 1–4 years are at the highest risk due to limited motor coordination and underdeveloped swimming capabilities (6–8). Behavioral elements such as insufficient supervision are also strongly implicated in the etiology of drowning events (8).

Epidemiological studies consistently report a male predominance in drowning incidents, with approximately 60% of victims being boys—possibly due to higher exposure to risk-taking behaviors and aquatic environments (9, 10). Socioeconomic disparities further contribute to drowning risk, as unequal access to water safety education, trained supervision, and timely medical intervention disproportionately affect vulnerable populations (11).

Accurate identification of prognostic factors following pediatric drowning is critical not only for improving survival rates but also for optimizing long-term neurological and functional outcomes in survivors (4). Prognostic indicators have traditionally been grouped into three domains: event-related clinical and environmental conditions (e.g., submersion time, water temperature), resuscitative efforts (timing and quality of cardiopulmonary resuscitation), and patient-specific factors (e.g., age, comorbidities) (3). In particular, the duration of hypoxia and delays in initiating life support have emerged as key determinants of neurological outcome (12).

Neurological assessment tools such as the Glasgow Coma Scale (GCS) and the Szpilman classification have shown prognostic utility in post-drowning evaluation, with low GCS scores and higher Szpilman grades correlating with adverse outcomes (13). Similarly, initial blood gas analysis—specifically, low arterial pH, elevated lactate, and high pCO₂ levels—has been associated with poor prognosis in pediatric cohorts (14, 15). Radiological evaluation, particularly chest radiography, can also reveal pulmonary infiltrates indicative of aspiration-related injury and has been linked to increased mortality in certain series (16, 17).

This study aims to provide a comprehensive clinical profile of pediatric drowning cases presented to a referral emergency department in Türkiye over a 13-year period. Specifically, the study examines demographic variables, clinical presentation, laboratory and radiological findings, emergency interventions, and short-term outcomes, with a particular focus on identifying variables associated with in-hospital mortality.

## Methods

### Study design, setting, and ethical considerations

This retrospective, cross-sectional study was conducted at a referral emergency department in Türkiye and included all pediatric patients admitted for seawater drowning between July 2011 and July 2024. Drowning cases were identified via the institutional Hospital Information Management System (HIMS), and all eligible cases were included regardless of data completeness. All eligible cases were included regardless of data completeness, and no exclusion criteria were applied based on variable availability. This full-case inclusion approach ensured a comprehensive census over the 13-year period.

Ethical approval was obtained from the Sinop University Local Ethics Committee (Decision No: 196; April 2025), and the study was conducted in accordance with the principles of the Declaration of Helsinki. Due to the retrospective nature of the study and the use of anonymized data, the requirement for informed consent was waived.

The primary objective of this study was to describe the demographic, clinical, and radiological features of pediatric drowning cases admitted to the ED. The secondary objective was to compare survivors and non-survivors in terms of laboratory parameters and resuscitation-related variables, with the aim of identifying potential indicators associated with mortality.

To minimize selection and information bias, all institutional cases meeting the diagnostic criteria were included without restriction. Dual-source data verification and independent double-entry were employed to enhance internal consistency and reliability.

In line with international standards, this study adopts the drowning definitions and data reporting guidelines set forth by the World Health Organization and the Utstein-style consensus (2). Cases were categorized as fatal or non-fatal, and core variables—such as demographics, prehospital response, clinical presentation, and outcomes—were collected and reported in accordance with these standardized frameworks.

In order to contextualize emergency care delivery, it is important to note that all patients in this study were managed within Türkiye's national Emergency Medical Services (EMS) system. Ambulances were equipped with advanced life support (ALS) capabilities and staffed by paramedics and emergency medical technicians (EMTs). Although physicians supervised EMS remotely, they were not present on scene. In the study region, average EMS response times typically remained under 10 min during the summer season.

### Study population and data collection

Eligible patients were defined as individuals aged 0–18 years who presented to the ED with a clinical diagnosis of drowning, confirmed through electronic medical records and ED documentation. Data were extracted retrospectively using a standardized collection form. Demographic variables included age, sex, and province of residence. Temporal characteristics such as month and time of presentation were recorded to explore seasonal patterns.

Clinical data included outcome status (discharged, deceased, transferred), hospital length of stay (ward or ICU), and presence of a family member involved in the same drowning incident. Emergency department disposition was categorized as discharge, ward admission, ICU admission, referral to an external ICU, or ED mortality.

Prehospital and in-hospital interventions were recorded, including cardiopulmonary resuscitation (CPR) administration (by EMS and/or in the ED) and endotracheal intubation. Neurological status was assessed on arrival using the Glasgow Coma Scale (GCS), and clinical severity was graded using the Szpilman score.

Radiological imaging reports were reviewed to identify pulmonary or neurological abnormalities that were interpreted by the treating clinicians as potentially attributable to the drowning incident. Imaging modalities included posteroanterior chest radiography, chest computed tomography (CT), and brain CT. Laboratory parameters included arterial blood gas values (pH, pCO₂, pO₂, HCO₃⁻, SO₂), pulse oximetry saturation, serum lactate, renal function tests (urea, creatinine), liver enzymes (AST, ALT, LDH), muscle injury markers (CK), and electrolytes (Na⁺, K⁺, Cl⁻).

### Statistical analysis

Statistical analyses were conducted using IBM SPSS Statistics version 25.0 (IBM Corp., Armonk, NY, USA). The normality of continuous variables was evaluated using the Shapiro–Wilk test (for sample sizes <50) or the Kolmogorov–Smirnov test (for ≥50), alongside visual inspection via Q–Q plots and histograms.

Descriptive statistics were used to summarize the data. Normally distributed continuous variables were presented as mean ± standard deviation (SD), while non-normally distributed variables were reported as median with interquartile range (IQR) and minimum–maximum values. Categorical variables were summarized as frequencies and percentages.

Comparisons between survivors and non-survivors were performed using the Mann–Whitney U test for continuous non-parametric variables, and Pearson's chi-square or Fisher's exact test for categorical variables, as appropriate. Exact *p*-values were reported for all inferential tests. Statistical significance was set at *p* < 0.05.

No data imputation was performed. Missing data were retained in the dataset and explicitly identified in the relevant tables. No subgroup, multivariate, or interaction analyses were conducted.

## Results

### Patient demographics and baseline characteristics

A total of 66 pediatric patients who experienced drowning and presented to the emergency department (ED) between July 2011 and July 2024 were included in the analysis. All cases identified as drowning in the hospital information system and aged 0–18 years were evaluated. No data imputation was performed; missing values were explicitly coded and retained for analysis.

The mean age of the patients was 10.64 ± 4.86 years, with a median of 10.93 years (range: 1.9–17.5). Males accounted for 60.6% (*n* = 40), and females for 39.4% (*n* = 26). The majority of cases occurred in July (53.0%) and August (39.4%). Of all patients, 46.97% were residents of Sinop and another 46.97% resided in other provinces. The overall hospital discharge rate was 77.3% (*n* = 51), and mortality occurred in 10.6% (*n* = 7). Most ED presentations occurred between 12:00 and 18:00 (74.2%). Regarding ED disposition, 37.9% of patients were discharged, 28.8% were admitted to the ward, 7.6% to the intensive care unit (ICU), and 13.6% were referred to external ICUs. Two patients died in the ED (Table 1).

**Table 1:** Demographic and clinical characteristics of pediatric drowning cases.

Parameter	Mean ± SD/n	Median (Min–Max)/%
Age	10.64 ± 4.86	10.93 (1.9–17.5)
Gender		
Male	40	60.60%
Female	26	39.40%
Month of Incident		
March	1	1.50%
June	3	4.50%
July	35	53.00%
August	26	39.40%
September	1	1.50%
Province of Residence		
Sinop	31	46.97%
Other	31	46.97%
No data	4	6.06%
Overall Outcome		
Deceased	7	10.60%
Discharged	51	77.30%
No data	8	12.10%
Time Interval of ED Presentation (hour)		
00:00–06:00	1	1.50%
06:00–12:00	2	3.00%
12:00–18:00	49	74.20%
18:00–24:00	14	21.20%
Disposition from ED		
Discharged	25	37.90%
Admitted to Ward	19	28.80%
Admitted to ICU	5	7.60%
Referred to External ICU	9	13.60%
Deceased (in ED)	2	3.00%
No data	6	9.10%
Drowning Incident Involving a Family Member		
Yes	18	27.27%
No	48	72.73%
Length of Stay in Ward		
1 day	12	63.16%
2 days	7	36.84%
Length of Stay in Intensive Care		
1 day	3	60.00%
2 days	1	20.00%
13 days	1	20.00%

ED, emergency department; ICU, intensive care unit.

In 27.3% of cases, at least one accompanying family member was also involved in the same drowning incident. Among patients admitted to the ward, 63.2% (*n* = 12) had a hospital stay of one day, and 36.8% (*n* = 7) stayed for two days. Of the five patients admitted to the ICU, three stayed for one day, one for two days, and one for thirteen days (Table 1).

### Clinical and laboratory parameters

Survivors and non-survivors were compared in terms of demographic, clinical, and laboratory characteristics. The median age was 11.00 years in survivors and 15.49 years in non-survivors (*p* = 0.043). The Glasgow Coma Scale (GCS) score was 15 in all survivors and 3 in all non-survivors (*p* < 0.001). The median Szpilman score was 1 in survivors and 6 in non-survivors (*p* < 0.001) (Table 2).

**Table 2:** Comparison of clinical and laboratory characteristics between survivors and non-survivors .

Parameter	Discharged (n)	Median (Q1–Q3) [Min–Max]	Deceased (n)	Median (Q1–Q3) [Min–Max]	Z	*p*-value	Missing Data *n* (%)
Age (years)	51	11 (5.76–15.08) [1.9–17.5]	7	15.49 (13.13–17) [6.7–17.3]	–2.017	0.043	8 (12.1%)
Glasgow Coma Scale (GCS)	50	15 (15–15) [8–15]	7	3 (3–3) [3–3]	–6.432	<0.001	9 (13.6%)
Spilman Score	49	1 (1–2) [0–4]	7	6 (5–6) [5–6]	–4.682	<0.001	10 (15.2%)
pH	48	7.32 (7.27–7.37) [7.053–7.52]	6	6.57 (6.53–6.81) [6.51–6.85]	–3.966	<0.001	12 (18.2%)
PCO₂ (mmHg)	48	42.8 (37.75–45.68) [21.8–59.1]	6	122.3 (94.7–149.78) [92.3–168]	–3.964	<0.001	12 (18.2%)
HCO₃ (mmol/L)	48	20.75 (18.6–23) [12.9–25.8]	6	12.2 (9.98–17.03) [8.4–18.6]	–3.400	<0.001	12 (18.2%)
SO₂ (%)	47	62.5 (53.9–86.6) [18.8–99.2]	6	28.15 (17.95–44.05) [5.8–86.5]	–2.779	0.004	13 (19.7%)
Pulse Oximetry SO₂ (%)	35	98 (96–98) [50–100]	6	25 (15–52.5) [0–90]	–3.815	<0.001	25 (37.9%)
Lactate (mmol/L)	30	3.93 (2.6–5.68) [0.9–13.5]	4	20.35 (13.1–26.79) [12.62–27]	–3.154	<0.001	32 (48.5%)
Urea (mg/dL)	46	24.5 (20.75–28) [10–38]	6	26 (19.75–33.25) [19–34]	–0.646	0.532	14 (21.2%)
Creatinine (mg/dL)	46	0.63 (0.54–0.75) [0.36–1.18]	6	1.07 (0.8–1.23) [0.74–1.26]	–3.340	<0.001	14 (21.2%)
AST (U/L)	46	25.5 (19–31) [13–264]	6	45 (28–276.5) [25–749]	–2.653	0.006	14 (21.2%)
ALT (U/L)	46	15.5 (11.75–20) [5–74]	6	33 (22–275.5) [19–583]	–3.328	<0.001	14 (21.2%)
LDH (U/L)	45	285 (236.3–327.5) [176–1,044]	6	359.65 (288–717.58) [279–1,688]	–2.310	0.019	15 (22.7%)
CK (U/L)	44	171.5 (123–209.5) [58–4,267]	6	152 (112.5–179.5) [105–238]	–0.836	0.418	16 (24.2%)

Clinical and laboratory variables were compared between survivors and non-survivors using the Mann–Whitney U test. Results are shown as median (Q1–Q3) [min–max]. Only non-parametric variables are included.

Non-survivors had significantly lower arterial pH and bicarbonate levels, and higher partial pressure of carbon dioxide (pCO₂) (*p* < 0.001 for all). Arterial oxygen saturation and pulse oximetry values were also significantly lower in non-survivors (*p* = 0.004 and *p* < 0.001, respectively). Lactate levels were significantly elevated in non-survivors (*p* < 0.001) (Table 2).

Serum creatinine, aspartate aminotransferase (AST), alanine aminotransferase (ALT), lactate dehydrogenase (LDH), and potassium levels were significantly higher in non-survivors (*p* < 0.05 for all). Sodium levels were also significantly higher in non-survivors (*p* < 0.001). No statistically significant differences were observed in creatine kinase (CK) or chloride levels (Table 2).

### Emergency interventions and radiological findings

Prehospital CPR was performed in 5 of 7 non-survivors (71.4%) and in none of the survivors (*p* < 0.001). In the ED, CPR was administered in all non-survivors (*n* = 7, 100%) and in none of the survivors (*p* < 0.001). Endotracheal intubation in the ED was performed in 7 of 7 non-survivors (100%) and in 1 of 51 survivors (2%) (*p* < 0.001). No patients underwent prehospital intubation (Table 3).

**Table 3:** Comparison of emergency interventions between survivors and non-survivors .

Variable	Survivors *n* (%)	Non-Survivors *n* (%)	*χ*²	*p*-value	Missing Data *n* (%)
Prehospital CPR (by EMS)			39.148	<0.001	9 (13.6%)
No	50 (100%)	2 (28.6%)			
Yes	0 (0%)	5 (71.4%)			
CPR in Emergency Department			57.000	<0.001	9 (13.6%)
No	50 (100%)	0 (0%)			
Yes	0 (0%)	7 (100%)			
Prehospital Intubation (by EMS)			–	–	9 (13.6%)
No	50 (100%)	7 (100%)			
Intubation in Emergency Department			48.878	<0.001	9 (13.6%)
No	49 (98%)	0 (0%)			
Yes	1 (2%)	7 (100%)			

Emergency interventions were compared between survivors and non-survivors using the Chi-square test with exact *p*-values. Data are presented as number (percentage). CPR, cardiopulmonary resuscitation; EMS, emergency medical services.

Lesions were observed on chest x-ray in 2 of 6 non-survivors (33.3%) and 3 of 50 survivors (6%) (*p* = 0.084). Among those who underwent chest computed tomography (CT), lesions were found in 2 of 6 non-survivors (33.3%) and 6 of 50 survivors (12%) (*p* = 0.200). Cranial CT demonstrated lesions in 1 of 6 non-survivors (16.7%) and in none of the 50 survivors (*p* = 0.107) (Table 4).

**Table 4:** Comparison of radiological findings between survivors and non-survivors .

Radiological Finding	Survivors *n* (%)	Non-Survivors *n* (%)	χ²	*p*-value	Missing Data *n* (%)
Lesion on Chest x-ray			4.922	0.084	10 (15.2%)
No	47 (94%)	4 (66.7%)			
Yes	3 (6%)	2 (33.3%)			
Lesion on Chest CT			1.991	0.200	10 (15.2%)
No	44 (88%)	4 (66.7%)			
Yes	6 (12%)	2 (33.3%)			
Lesion on Brain CT			8.485	0.107	10 (15.2%)
No	50 (100%)	5 (83.3%)			
Yes	0 (0%)	1 (16.7%)			

Radiological findings were compared between survivors and non-survivors using the Chi-square test with exact *p*-values. All values are presented as number (percentage). CT, computed tomography. Chest x-ray refers to standard posteroanterior (PA) projection.

## Discussion

This study provides one of the most detailed and up-to-date analyses of pediatric drowning cases in Türkiye, encompassing a 13-year period and integrating clinical, biochemical, radiological, and outcome-based parameters. Among 66 pediatric patients evaluated, the overall in-hospital mortality rate was 10.6%, with statistically significant associations observed between mortality and variables such as Glasgow Coma Scale (GCS) score, Szpilman score, arterial blood gas parameters (pH, lactate, pCO₂, bicarbonate), and key biochemical markers including creatinine, AST, ALT, LDH, sodium, and potassium. Furthermore, the finding that 27.3% of cases involved at least one accompanying family member highlights the social and behavioral dimensions of these incidents, including bystander rescue attempts and clustered exposure patterns. These findings emphasize the critical need for early risk stratification, rapid emergency response, and preventive strategies tailored to seasonal and demographic risk profiles.

Consistent with previous literature, male sex was predominant (60.6%) among pediatric drowning cases, which aligns with global data identifying male sex as a significant risk factor (10). Although the mean age in our cohort was approximately 10.6 years, children under 5 years accounted for nearly one-fifth of cases (19.6%). This group, widely recognized as highly vulnerable due to limited motor coordination, insufficient swimming ability, and exploratory behavior, has been repeatedly shown to be at heightened risk for fatal drowning (6–8). Interestingly, our study reported no mortality in this age group, a finding that may reflect protective parenting behaviors prevalent in the local context. In contrast, a higher proportion of fatalities occurred among adolescents aged 15–18 years, suggesting behavioral and environmental risk exposure increases with age. However, age was not independently associated with mortality, likely due to the limited number of fatal cases.

The strong seasonal clustering of cases, with over 90% occurring in July and August, underscores the relationship between drowning incidents and tourism-related increases in coastal activity. Approximately half of the patients were non-residents of Sinop, further reinforcing the impact of seasonal tourism. The majority of ED presentations occurred between 12:00 and 18:00, a time window associated with peak beach usage and reduced adult supervision, as also observed in previous studies (9).

The involvement of accompanying family members in over one-quarter of cases reflects a recurring theme in drowning literature: the risk of multi-victim incidents during rescue attempts. Prior research from Türkiye reported that in nearly half of such attempts, rescuers themselves died (18). This highlights the urgent need for public education regarding safe rescue protocols and structured water safety training, particularly for caregivers and adolescents.

The mortality rate observed in our study (10.6%) is somewhat lower than rates reported in similar pediatric cohorts globally. For instance, Güzel et al. (2013) reported a mortality rate of ∼20% (5), while a nationwide analysis from Thailand found pediatric drowning mortality to be 12.6% (19). Variations may be explained by differences in emergency response times, availability of resuscitation, geographic factors, and population behavior.

As expected, neurological status at admission was strongly associated with outcome. All non-survivors had a GCS score of 3 and a median Szpilman score of 6, compared to a GCS of 15 and Szpilman score of 1 among survivors—findings consistent with prior reports underscoring the prognostic value of these clinical scores (20, 21). Laboratory markers further corroborated poor perfusion and multi-organ dysfunction in non-survivors. Severe metabolic derangements such as marked acidosis (mean pH 6.57), hypercapnia (pCO₂ 122.3 mmHg), elevated lactate (20.35 mmol/L), and low bicarbonate (12.2 mmol/L) were observed. These findings are in line with previous studies showing that pH < 7.0, lactate ≥ 14 mmol/L, and bicarbonate < 15 mmol/L have been strongly associated with mortality or severe neurological outcomes (21, 22).

Similarly, elevations in AST, ALT, and LDH have been consistently associated with adverse outcomes in pediatric drowning (21, 23). Our study also found significantly higher serum sodium and potassium concentrations in non-survivors, both of which are markers of cellular injury and water-electrolyte imbalance in drowning victims.

The laboratory abnormalities observed in non-survivors are mechanistically coherent with the final common pathway of drowning—hypoxaemia and tissue hypoperfusion (4, 24). Irrespective of the drowning medium, alveolar flooding and the washout and inactivation of surfactant impair gas exchange and produce ventilation–perfusion mismatch, causing carbon-dioxide retention (high pCO₂) and, together with circulatory arrest, profound tissue hypoxia (4, 24). Anaerobic glycolysis then generates lactate, driving a mixed metabolic and respiratory acidosis with markedly low pH and consumption of bicarbonate (low HCO₃⁻)—a pattern repeatedly associated with poor outcome after submersion (21, 22, 25). The same hypoxic-ischaemic insult and hypoperfusion, sometimes compounded by rhabdomyolysis, can precipitate acute kidney injury via ischaemia–reperfusion and acute tubular necrosis, with rising creatinine (26, 27), while hepatocellular and generalized cellular injury release transaminases (AST, ALT) and lactate dehydrogenase, the latter a non-specific marker of tissue damage and haemolysis; such multi-organ involvement has been systematically documented after pediatric drowning (26) and mirrors the enzyme elevations reported in earlier prognostic series (21, 23). The higher sodium and potassium concentrations in non-survivors are consistent with aspiration of hypertonic seawater and with transcellular potassium shift and cellular necrosis in the setting of severe acidosis and hypoxia. It should be emphasized, however, that the volume of fluid aspirated by most drowning victims (typically only 3–4 mL/kg) is too small to produce the classic electrolyte and fluid-compartment shifts once attributed to salt- versus freshwater drowning (4); the marked derangements observed in our fatal cases therefore most likely reflect larger-volume aspiration together with more severe systemic hypoxic injury rather than a medium-specific effect. Taken together, these markers are best interpreted collectively as indices of hypoxic burden and multi-organ dysfunction rather than as independent processes (4, 24, 26).

Although radiographic findings did not reach statistical significance, lesions on posteroanterior chest x-ray were more common in non-survivors (33.3% vs. 6%). Prior studies have suggested that early radiographic abnormalities—often indicative of aspiration-related pulmonary edema—are associated with higher rates of clinical deterioration and worse outcomes (16, 17, 28, 29). Our findings, although limited by sample size, suggest a similar trend and warrant further investigation in larger cohorts.

The need for resuscitation and intubation was also strongly associated with mortality. All non-survivors received CPR both prehospitally and in the ED, and all were intubated upon arrival. In contrast, none of the survivors required CPR, and only one survivor (2%) underwent intubation. These findings mirror previous literature demonstrating that the need for advanced airway management and prolonged CPR are robust indicators of poor outcome (21). Timely and effective prehospital intervention—especially Basic Life Support—remains critical in altering the trajectory of submersion injuries in children.

Because only seven deaths occurred and no multivariable analysis was feasible, the variables reported here should be interpreted as factors associated with mortality on univariate testing rather than as independent predictors; with so few events, these associations are vulnerable to type I error and confounding and require confirmation in adequately powered, multicentre studies.

Drowning outcome is governed chiefly by the magnitude and duration of hypoxia (4, 24). Submersion (downtime) duration is the strongest single determinant of survival and neurological recovery: in a systematic review and meta-analysis of scene factors, it was the only robust, independent predictor of outcome, with favourable outcomes becoming progressively less likely as submersion time lengthened and submersions beyond 25 min almost uniformly fatal (3). The rapidity and quality of early resuscitation—particularly immediate bystander basic life support before EMS arrival—can substantially modify that trajectory; bystander CPR has been associated with a nearly threefold increase in neurologically favourable survival after drowning-related cardiac arrest (24, 30), and both prehospital conditions and subsequent hospital care shape the ultimate prognosis (31). The type of water is now considered clinically less important than once thought: although seawater is hypertonic and freshwater hypotonic, the volumes aspirated are typically small, and the decisive events in both are surfactant washout and inactivation, alveolar collapse and hypoxaemia rather than the historically emphasized electrolyte or fluid shifts (4, 24); the volume aspirated matters mainly insofar as it worsens gas exchange. In our cohort, downtime and time-to-CPR could not be quantified from the records (see Limitations), so several of the abnormalities we report are best understood as downstream surrogates of these primary determinants.

Our study addresses clinical risk stratification in living children, which is conceptually distinct from the forensic problem of confirming drowning at autopsy—a diagnosis of exclusion with no single pathognomonic finding. Recent prospective comparative studies have advanced objective post-mortem markers for this purpose: the Drowning Index discriminated freshwater drowning from hanging and sudden cardiac deaths (32) and, in a subsequent comparison, the Pleural Effusion Ratio outperformed the Drowning Index for diagnosing freshwater drowning (33). Although these indices are diagnostic rather than prognostic and derive from freshwater series, they illustrate how quantitative, reproducible metrics can complement conventional assessment, and the development of analogous objective markers for the living patient is a worthwhile direction for future clinical–forensic collaboration.

### Limitations

This study has several limitations inherent to its retrospective and single-center design. First, certain clinical variables and long-term outcomes could not be retrieved, particularly for patients referred to external institutions, limiting the assessment of neurological sequelae and functional recovery. Second, the relatively small number of fatal cases may have reduced the statistical power to detect differences in some subgroup analyses. Third, although imaging and laboratory parameters were interpreted using standardized protocols, the retrospective nature of data extraction may have introduced observer variability. Additionally, all drowning incidents occurred in a seawater setting, which limits the generalizability of the findings to freshwater or swimming pool drownings, where pathophysiological and clinical profiles may differ. Lastly, the findings are specific to a coastal region of Türkiye and may not fully reflect drowning patterns in other geographic or healthcare contexts.

Given that only seven deaths occurred, the study was statistically underpowered for multivariable modelling, and all comparisons are necessarily univariate. With so few events, the observed associations may be unstable, are vulnerable to type I error arising from multiple comparisons, and cannot be adjusted for confounding; they should therefore be regarded as exploratory and hypothesis-generating rather than as confirmed prognostic relationships, and require validation in larger, multicentre cohorts.

Missing data were considerable for several parameters (for example, lactate 48.5%, pulse oximetry saturation 37.9%, and arterial blood gas variables up to 18%–20%), reflecting the retrospective design and the fact that the most severely affected children were often resuscitated or transferred before a complete panel could be obtained. No imputation was performed and analyses were restricted to available cases. Because measurements were more likely to be complete in severely ill patients, the data are unlikely to be missing completely at random, and complete-case analysis may have over- or under-estimated the magnitude of some associations.

Importantly, submersion (downtime) duration and the interval from submersion to the initiation of basic life support—arguably the most influential determinants of survival and neurological outcome after drowning—were not consistently documented in the prehospital and emergency records and could not be analysed. Several of the clinical and laboratory differences we observed (for example, the degree of acidosis and hyperlactataemia) are themselves surrogates of hypoxic burden and submersion time, so residual confounding by these unmeasured variables is likely and the reported associations cannot be assumed to be independent of them.

Moreover, the retrospective design may have introduced selection and information bias, and the absence of standardized data for all cases could limit the precision of some estimates. The direction and magnitude of such bias are uncertain but may have led to underestimation or overestimation of some associations.

## Conclusion

In this 13-year single-centre series of pediatric seawater drowning, outcome was decided largely at the front door: every non-survivor arrived with a Glasgow Coma Scale of 3, a high Szpilman grade, and severe metabolic derangement, and every non-survivor required cardiopulmonary resuscitation and intubation, whereas none of the survivors did. A depressed neurological status, a high Szpilman score, and a simple bedside panel—arterial pH, lactate, bicarbonate, and creatinine—therefore give clinicians an immediate, low-cost means of identifying the child at highest risk and mobilising critical-care resources without delay. These associations were derived from univariate analysis of a small number of deaths and should be regarded as factors associated with, rather than independent predictors of, mortality; confirmation in larger multicentre cohorts that also capture submersion time and time-to-CPR is needed. Equally striking is the epidemiological signature of these events: most cases clustered in July and August, nearly half involved visiting families, and more than a quarter involved a second person in the same incident—a pattern that points to concrete, seasonally targeted prevention, including lifeguard coverage at peak beach hours, water-safety education for tourists and adolescents, and training in safe rescue to prevent bystander deaths. Coupling early clinical risk stratification with region-specific prevention offers the most realistic route to reducing childhood drowning mortality in coastal, tourism-driven settings.

Future efforts should focus on multicenter prospective studies, development of region-specific drowning risk models, and implementation of scalable preventive interventions—particularly in coastal regions with high seasonal tourist influx. Integration of standardized reporting frameworks and improved inter-institutional data sharing may further enhance national drowning surveillance and inform public health policy.

## Data Availability

The raw data supporting the conclusions of this article will be made available by the authors, without undue reservation.
